# Factors associated with use of wearable technology to support activity, well-being, or a healthy lifestyle in the adult population and among older adults

**DOI:** 10.1371/journal.pdig.0000245

**Published:** 2023-05-10

**Authors:** Maiju Kyytsönen, Tuulikki Vehko, Heidi Anttila, Jonna Ikonen

**Affiliations:** 1 Health and Social Service System Research, Finnish Institute for Health and Welfare, Helsinki, Finland; 2 Functioning and Service Needs, Finnish Institute for Health and Welfare, Helsinki, Finland; 3 Monitoring, Finnish Institute for Health and Welfare, Helsinki, Finland; Tsinghua University, CHINA

## Abstract

The use of wearable technology, which is often acquired to support well-being and a healthy lifestyle, has become popular in Western countries. At the same time, healthcare is gradually taking the first steps to introduce wearable technology into patient care, even though on a large scale the evidence of its’ effectiveness is still lacking. The objective of this study was to identify the factors associated with use of wearable technology to support activity, well-being, or a healthy lifestyle in the Finnish adult population (20–99) and among older adults (65–99). The study utilized a cross-sectional population survey of Finnish adults aged 20 and older (n = 6,034) to analyse non-causal relationships between wearable technology use and the users’ characteristics. Logistic regression models of wearable technology use were constructed using statistically significant sociodemographic, well-being, health, benefit, and lifestyle variables. Both in the general adult population and among older adults, wearable technology use was associated with getting aerobic physical activity weekly according to national guidelines and with marital status. In the general adult population, wearable technology use was also associated with not sleeping enough and agreeing with the statement that social welfare and healthcare e-services help in taking an active role in looking after one’s own health and well-being. Younger age was associated with wearable technology use in the general adult population but for older adults age was not a statistically significant factor. Among older adults, non-use of wearable technology went hand in hand with needing guidance in e-service use, using a proxy, or not using e-services at all. The results support exploration of the effects of wearable technology use on maintaining an active lifestyle among adults of all ages.

## Author summary

Wearable technology is increasingly used to support health and well-being. At the same time, the markets are expanding, and the healthcare sector is becoming aware of the possible benefits. In our study, we looked at wearable technology use from a population survey perspective. The analysis demonstrated that wearable technology use was more common among 20-54-year-olds and that the users reached the recommended amount of aerobic physical activity weekly more often than non-users. The increased activity level could impact public health in a positive way. However, since the data set was cross-sectional, we do not know if the increased activity level is a consequence of wearable technology use or were the wearable technology users already more active before starting to use the technology. Other insights were that wearable technology use decreases after the age of 54 and that among older adults wearable technology use is strongly associated with independent e-service use. We hope this study encourages conducting more research on WT use and that the results inspire the design of products also for older adults who are not familiar with using this kind of modern technology.

## Introduction

The use of wearable technology (WT) and the devices’ mobile health applications have become common in Western countries as people are increasingly interested in their own health and well-being. Many see WT as a tool for improving health and well-being and a way to receive information about the body and about oneself in general [1,2]. Fitness and physical activity or positions (for example, sitting versus standing) have been identified as the most desired dimensions for self-tracking devices among typical consumers. Other possible tracking dimensions include stress level, mood or feelings, sleep patterns, location, weight and diet, general vital signs, and disease or disorder symptoms [3].

The reliability of the information provided by WT sensors varies depending on the technological solutions, situational factors, and physical characteristics, such as hand movement, walking speed, sleep disturbances or morbid obesity [4–7]. On the other hand, instead of viewing WT as simply a tool for measuring activity, sleep, bodily functions, or behavioural patterns, it can be seen as a way of changing the possibilities and conditions of how a person can know oneself [2] and how they act [8]. For example, educators have identified in situ contextual information, communication options, and first-person view as affordances for WT use [9]. Furthermore, many WT devices can provide information on long-term trends in measurements, which give insight into the well-being of a person over a longer period [5]. This habitual data can be used in the healthcare context [10].

WT covers a wide range of different devices, including conventional smartwatches and smart jewellery that can be worn on the finger, ear, or neck [10]. The users’ reasons for adopting WT have been shown to differ from device to device and from group to group [11]. Some identified reasons that influence adoption of WT for fitness indirectly and directly are the compatibility of the technology with users and their lifestyle, the innovativeness of the device, the effort required to use it, social influence, and habit. Another significant factor for adoption is how effective the users expect the device to be for example in reducing health-related threats [12]. However, a study conducted in a lower socioeconomic community indicated that those who adopted a new WT were more likely to already lead a healthier lifestyle [13]. Identified reasons for discontinuing WT use include loss of motivation, perceived measurement issues, poor design, obsessive tracking, and privacy concerns [14]. Privacy concerns seem to be a major issue when potential users even consider WT use [9,11].

WT has been acknowledged in the context of healthcare, where steps are being slowly taken to introduce the use of WT and applications into patient care [10,15] and to support independent living in the ageing population [16]. In healthcare, the possibilities of using WT for predicting clinical outcomes have been studied [17]. Other fields of applications include monitoring the activity level of patients with chronic obstructive pulmonary disease [18], predicting patient-reported pain scores [19], arrhythmia monitoring [7] and assessing the worsening of dementia symptoms [20]. Furthermore, general practitioners have indicated interest in the option of prescribing mHealth apps and devices to their patients [21]. Still, there seems to be a lack of standard guidelines for the clinical study of WT [22] and the studies on WT in the healthcare context call for further validation and evidence of effectiveness before it is possible to introduce WT in medicine and patient care in a more extensive scale [7,10,15,17–19,21].

WT users have shown interest in the option of sharing the information provided by their WT device with a healthcare professional [3,23–24]. Even though the validation of WT’s effectiveness is demanding, the introduction of WT into patient care has long been anticipated to bear great potential [15,25–26]. However, while the functions of WT for medical purposes can be overlapping with fitness or well-being use, medical WT may call for a specific type of measuring. For example, posture-related medical WT may be designed to detecting falls instead of detecting sitting versus standing. [15] In the European Union, WT for patient care can be distinguished from fitness WT with a CE Mark [27]. Products used for patient care need to be recognized as medical devices, which is a long process that requires multiple appraisals. On top of that, a separate validation process is recommended for healthcare WT [15], which can be done using the Digi-HTA framework. The framework supports the health technology assessment process for digital healthcare services [28]. Researchers have suggested that collecting device requirements from users and physicians would be beneficial for designing WT [22] and that collecting feedback of case examples from clinicians could help develop especially assistive technology for vulnerable groups [29]. Feedback from users and healthcare professionals could for example help product designers in generating an apprehensible summary view of the data.

While WT use is common in Finland [30], it is not known who the WT users are. Prior international research has recruited participants from social media or other web-based platforms [8,14,22], examined an otherwise restricted sample [12–13,31], for example consisting only of students and employees with graduate-level education and mid-to-high incomes or the study sample has had a specific age limit [1,3,31–33]. One study did have a large population, but it did not entirely represent the intended population in terms of sex, age, education, and body mass index (BMI) [33]. To broaden scientific knowledge, conducting research on WT in large, heterogenous study samples has been recommended [13,31], especially researching older adults’ technology usage with representative study samples [34].

On top of not knowing who the users are from a population perspective, WT use has been suggested as one solution for maintaining health in the ageing population [16,22]. Therefore, this study aimed to identify which factors are associated with use of WT, that is used to support activity, well-being, or a healthy lifestyle, in the Finnish adult population (20–99) and among older adults (65–99) utilizing a population survey data set. The object of this study was to construct logistic regression models using statistically significant sociodemographic, well-being, health, benefit, and lifestyle variables. A diverse range of independent variables was selected, since the increasing use of digital devices gives rise to new phenomena, which make earlier established scales concentrating on for example (computer) attitude insufficient [35].

## Results

The use of WT supporting activity, well-being or a healthy lifestyle was common; 28% of the population used it (1,692/6,034, 95% confidence interval: 26.3–29.8). The prevalence of WT use was grouped by sociodemographic, well-being, health, benefit, and lifestyle factors (Table 1). Based on the prevalence and confidence intervals, sex, financial difficulties, and a BMI of 25 or over did not seem to be connected to WT use. Instead, age, education level, marital status, quality of life, state of health, mental well-being, physical activity, sleep, and a BMI of 30 and over seemed to be connected to WT use (Table 1).

**Table 1:** Prevalence (%) and 95% confidence intervals of wearable technology use in the adult population grouped by socio-demographic, well-being, benefit, and lifestyle factors.

Variable	Response category	Wearable technology use (%)	95% CI		n
			Lower	Upper	
**Socio-demographics**					
Sex (n = 6,034)	Male	26.2	23.6	29.0	2,768
	Female	29.6	27.3	32.0	3,266
Age group (n = 6,034)	20–34	36.4	31.8	41.3	1,359
	35–54	37.5	33.9	41.2	1,944
	55–64	27.4	24.1	30.9	986
	65–74	16.5	14.2	19.2	973
	>74	4.9	4.0	5.9	771
Education level (n = 5,873)	Low	25.1	22.5	28.0	2,470
	Middle	30.5	27.4	33.8	1,868
	High	32.3	28.9	35.9	1,535
Marital status (n = 5,980)	Married/ in a registered relationship	29.9	27.6	32.3	2,651
	Cohabiting	34.5	30.0	39.3	1,250
	Separated/ divorced/ widowed	18.9	15.4	22.9	895
	Single	25.0	20.8	29.7	1,184
Financial difficulties (n = 5,892)	Yes	29.1	24.5	34.1	1,030
	No	28.0	26.1	29.9	4,862
**Well-being**					
Quality of life (WHOQOL-8) (n = 5,985)	Better than average	31.1	28.8	33.5	3,339
	Poorer than average	24.6	22.1	27.4	2,646
State of health (n = 5,956)	Good/ fairly good	33.3	31.0	35.7	3,851
	Average/ poor	19.4	16.9	22.1	2,105
Mental well-being (SWEMWBS) (n = 5,620)	High/ average	30.4	28.4	32.5	4,434
	Possible/ probable depression	21.8	18.2	26.0	1,186
BMI > = 25 (n = 5,905)	Yes	27.4	25.3	29.7	3,530
	No	30.1	27.2	33.2	2,375
BMI > = 30 (n = 5,905)	Yes	25.1	21.6	28.9	1,311
	No	29.5	27.5	31.6	4,594
**Lifestyle**					
Adequate aerobic physical activity weekly (n = 5,004)	Yes	37.1	34.4	40.0	2,790
	No	20.3	17.8	23.1	2,214
Adequate aerobic and muscle-strengthening physical activity weekly (n = 4,766)	Yes	39.3	35.8	42.9	1,860
	No	24.5	22.2	27.0	2,906
Usually sleeps enough (n = 5,904)	Yes	27.3	25.3	29.3	4,477
	No	32.7	28.9	36.9	1,427
**Benefit**					
E-services help in maintaining a healthy lifestyle (n = 5,710)	Agree	34.2	30.6	38.0	1,449
	Disagree/ no opinion	27.5	25.4	29.6	4,261
E-services help in taking an active role (n = 5,668)	Agree	37.0	34.1	40.1	2,392
	Disagree/ no opinion	23.9	21.6	26.2	3,276

**Fig 1:**
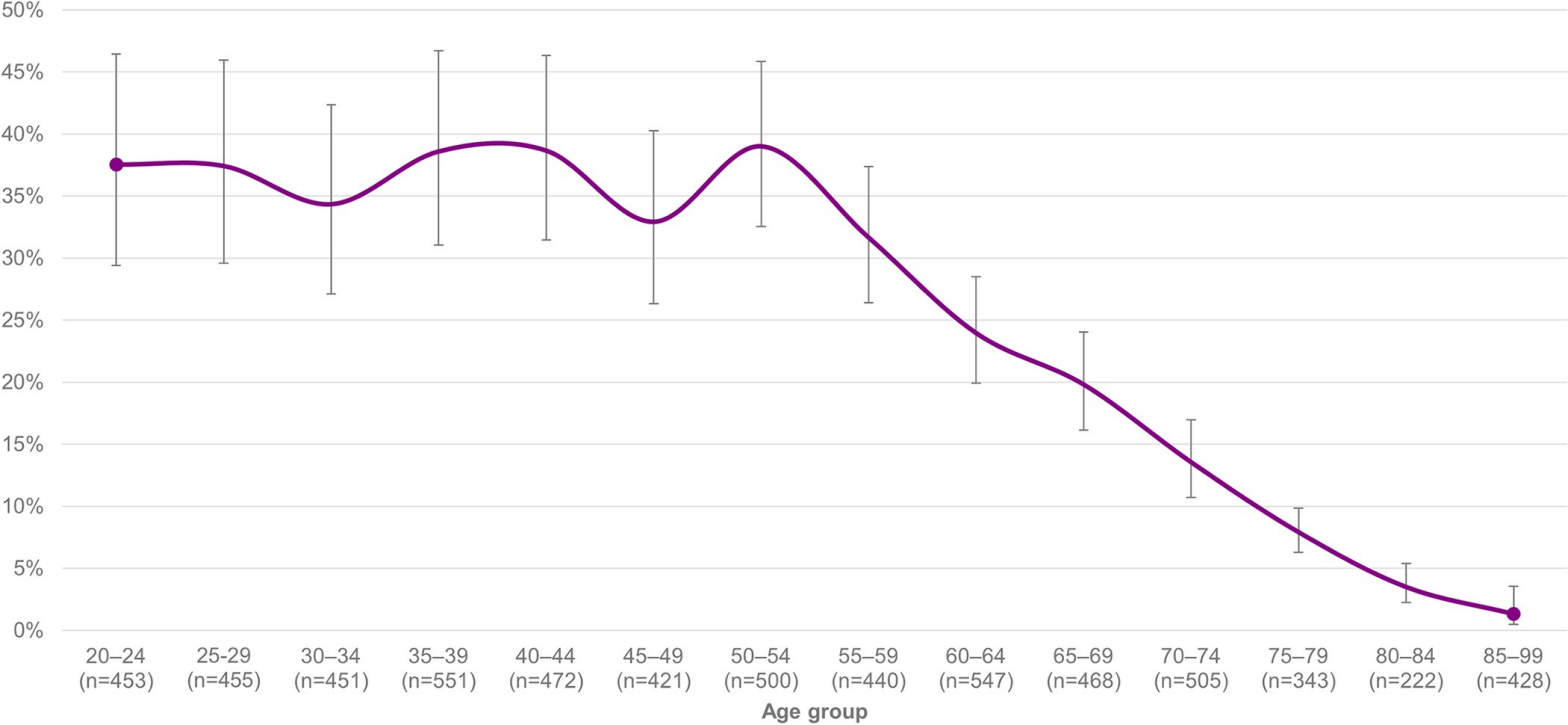
The prevalence of wearable technology use among different age groups and 95% confidence intervals of the estimates (N = 6,034).

The use of WT decreased after the age of 54 (Fig 1). However, the use of smart technology that supports independent living did not indicate a similar decrease with age: 1.4% (85/6,034, 95% CI: 1.0–1.9) of 20-99-year-olds used such technologies, while the percentage was 1.7 (30/1,744, 95% CI: 1.2–2.4) among 65-99-year-olds.

Regression models were constructed to analyse the independent associations of statistically significant variables in the adult population (Table 2). The first model included age group, education level, marital status, quality of life, state of health, mental well-being, aerobic physical activity, aerobic and muscle-strengthening physical activity, sleep, BMI, taking an active role in looking after ones’ own health and well-being and maintaining a healthy lifestyle with the help of e-services. The model selection was done by reviewing the results of the Wald test: not statistically significant variables were excluded (Table 2).

**Table 2:** Wald test p-values for binary logistic regression in Model 1 and Model 2.

	Wald test p-values, Model 1	Wald test p-values, Model 2
**Corrected Model**	**<0.001**	**<0.001**
**Intercept**	**<0.001**	**<0.001**
**Age group**	**<0.001**	**<0.001**
**Education level**	0.330	
**Marital status**	**0.010**	**0.001**
**Quality of life (WHOQOL-8)**	0.891	
**Good state of health**	**0.039**	**0.010**
**Mental well-being (SWEMWBS)**	0.574	
**Aerobic physical activity weekly**	**0.001**	**<0.001**
**Aerobic and muscle-strengthening physical activity weekly**	0.088	
**Usually sleeps enough**	**0.025**	**0.012**
**BMI > = 25**	0.601	
**BMI > = 30**	0.856	
**Electronic social welfare and healthcare services help me to maintain a healthy lifestyle**	0.799	
**Electronic social welfare and healthcare services help me to take an active role in looking after my own health and well-being**	**<0.001**	**<0.001**

The results of the logistic regression (Model 2) confirmed that the use of WT decreases in older age groups (Table 3). However, the confidence intervals of the age group of 35–54 varied on both side of 1 (no association) and since 20–34 was the reference group, the statistically significant decrease in use was noteworthy mainly for over 54-year-olds. Marital status was associated with WT use: those who were married or in a registered relationship used WT 1.96 times more often than singles. WT use demonstrated a slight association with a good state of health, but the lower level of the confidence interval was close to no correlation. WT use was associated with getting adequate aerobic physical activity weekly. Those who had adequate physical activity used WT 2.06 times more often than others. Usually sleeping enough demonstrated a slight negative association with WT use: those who usually slept enough used WT less often. Agreeing with the statement, ‘Social welfare and healthcare e-services help me to take an active role in looking after my own health and well-being’, was also associated with WT use. Those who agreed with the statement used WT 1.81 times more often than others (Table 3).

**Table 3:** Binary logistic regression results of Model 2 presented as odds ratios, 95% confidence intervals, p-values, and variance inflation factors.

		OR	95% CI		p	VIF
			lower	upper		
**Age group**						1.305
	20–34	**1.00**				
	35–54	**0.89**	0.65	1.22	<0.001	
	55–64	**0.53**	0.38	0.74	<0.001	
	65–74	**0.30**	0.22	0.43	<0.001	
	>74	**0.13**	0.09	0.20	<0.001	
**Marital status**						1.318
	Married or in a registered relationship	**1.96**	1.42	2.70	<0.001	
	Cohabiting	**1.62**	1.13	2.32	0.009	
	Separated, divorced, or widowed	**1.75**	1.14	2.68	0.011	
	Single	**1.00**				
**State of health**						1.156
	Good or fairly good	**1.37**	1.08	1.75	0.010	
	Average, fairly poor or poor	**1.00**				
**Adequate aerobic physical activity weekly**						1.086
	Yes	**2.06**	1.66	2.57	<0.001	
	No	**1.00**				
**Usually sleeps enough**						1.056
	Yes	**0.72**	0.56	0.93	0.012	
	No	**1.00**				
**Social welfare and healthcare e-services help me to take an active role in looking after my own health and well-being**						1.013
	Agree	**1.81**	1.47	2.22	<0.001	
	Disagree or no opinion	**1.00**				

A logistic regression analysis was additionally run for the population aged 65 or more (Table 4). The use of WT among older adults was associated with marital status, adequate aerobic physical activity weekly, and independent e-service use (Table 5). Those who were married, in a registered relationship (OR 5.77), cohabiting (OR 5.00), or separated, divorced, or widowed (OR 4.60) used WT more often than those who were single. Those older adults who got adequate aerobic physical activity weekly used WT 1.85 times more often compared to those who did not. Those older adults who used e-services independently used WT 15.39 times more often than those who needed guidance in e-service use, used a proxy or did not use e-services at all.

**Table 4:** Wald test p-values for binary logistic regression among older adults (aged 65 and over).

	Wald test p-values, Model 1	Wald test p-values, Model 2	Wald test p-values, Model 3
**Corrected Model**	**<0.001**	**<0.001**	**<0.001**
**Intercept**	**<0.001**	**<0.001**	**<0.001**
**10-year age groups**	0.261		
**Sex**	0.547		
**Education level**	0.509		
**Marital status**	**0.006**	**0.012**	**0.011**
**Quality of life (WHOQOL-8)**	0.868		
**Good state of health**	0.256		
**Mental well-being (SWEMWBS)**	0.072		
**Aerobic physical activity weekly**	**0.040**	**0.001**	**0.001**
**Aerobic and muscle-strengthening physical activity weekly**	0.193		
**Usually sleeps enough**	0.363		
**BMI > = 30**	0.534		
**Independent e-service use**	**0.000**	**<0.001**	**<0.001**
**Electronic social welfare and healthcare services help me to maintain a healthy lifestyle**	0.529		
**Electronic social welfare and healthcare services help me to take an active role in looking after my own health and well-being**	**0.035**	**0.062**	

**Table 5:** Binary logistic regression results of Model 3 for older adults (aged 65 and over) presented as odds ratios, 95% confidence intervals, p-values, and variance inflation factors.

		OR	95% CI		p	VIF
			lower	upper		
**Marital status**						1.043
	Married or in a registered relationship	5.77	2.01	16.58	0.016	
	Cohabiting	5.00	1.55	16.07	0.024	
	Separated, divorced, or widowed	4.60	1.53	13.88	0.690	
	Single	**1.00**				
**Adequate aerobic physical activity weekly**						1.008
	Yes	1.85	1.30	2.62	<0.001	
	No	**1.00**				
**Independent e-service use**						1.043
	Yes	15.39	7.90	30.00	<0.001	
	No	**1.00**				

## Discussion

This cross-sectional study aimed to identify which factors are associated with use of WT, that is used to support activity, well-being, or a healthy lifestyle, in the Finnish adult population (20–99) and among older adults (65–99). According to the results, WT use in the adult population and among older adults was positively associated with marital status and getting enough aerobic physical activity weekly. In the adult population WT use was also associated with younger age, while age among older adults was not a statistically significant factor. WT use in the general adult population was associated with usually not sleeping enough and agreeing with that social welfare and healthcare e-services help in taking an active role in looking after my own health and well-being. WT use among older adults was strongly associated with independent e-service use. The data collection was done during the first year of the COVID-19 pandemic, which as an exceptional time might have affected WT use and the physical activity level of people.

### General adult population

WT use decreased systematically with age so that 20-34-year-olds seemed to use WT markedly more often than those over 74 years of age, which is in line with previous studies [22,33]. The discovery is unfortunate because older adults (and the elderly) could especially benefit from monitoring their long-term measurement results. Monitoring these numerical trends could help in detecting undesired changes in well-being [5]. The data could also help in bringing these changes (e.g., in reduced sleep) to the attention of a doctor [3,24]. This kind of option has been designed in the context of Finland’s national medical record service for the citizens: My Kanta Pages, but its full potential can only be realized after a legislative transition period is over in January 2024.

WT use was more popular among persons who were married, in a registered relationship, cohabiting, separated, divorced, or widowed compared to being single. The association between WT use and being in a relationship might be due to those in a relationship having a higher motivation to take care of one’s own health and well-being since loneliness has previously been shown to strongly associate with decreased physical activity [36]. From another point of view, attracting a partner has been identified as a key motivator for physical activity among single men [37]. This finding cannot however be supported by our study findings that take a wider view of the population.

Previously, technology-enhanced interventions have been recognized as one solution for motivating people to lead a healthier life [16,25,38]. In this study, a good or fairly good self-assessed state of health showed a slight positive association with WT use. On the other hand, it is possible that people who already lead a healthier life and are healthier start using WT more often [13]. According to our results, getting adequate aerobic physical activity weekly was associated with WT use, which is in line with previous intervention studies [25–26,38] and a literature search that suggested a positive relationship existed between chronic obstructive pulmonary disease patients’ physical activity and WT use, even though more evidence was needed to confirm it [18].

The results indicated that those who do not usually sleep enough use WT slightly more often than those who do get enough sleep. The discovered association can be an actual difference between the groups. Another possible explanation is that WT users receive sensor data on their sleep, which gives them a more realistic (numerical) picture of their day-to-day sleep, making it easier to keep track of the current situation. A study on diabetes patients provides slight evidence to support this speculation: the subjective evaluations of WT users correlated only moderately with sleep sensor data, while a clear positive correlation was found between physical activity sensor data and subjective evaluation [39]. At the same time, an interview study discovered that while WT users receive data on their sleep, they did not have the means of taking action to improve their sleep [40].

Our study found a positive association between WT use and agreeing with the statement that social welfare and healthcare e-services help in taking an active role in looking after one’s own health and well-being. It seems that WT use can potentially act as a mediator in the attitudinal change from people being the target of interventions to being agents of their own health and well-being. This shift has been identified in the larger picture of the digital health field, where the emergence of empowered customers calls for personalised care that can better support individuals’ health and well-being instead of directing people to care pathways designed beforehand [41]. As a downside, researchers have suggested that feedback provided by WT devices can cause dependency which leads to decreased motivation to engage in physical activity when the device is not available. As a solution, the design of the devices should facilitate the intrinsic motivation, autonomy, and self-determination of the users. [8]

### Statistically non-significant factors

The large-scale population survey made it possible to explore the significance of a wide range of independent variables and compare the findings to other studies that have analysed a more limited number of variables. In our analysis, the following variables seemed non-significant for WT use: sex, financial difficulties, BMI, education level, quality of life, mental well-being, adequate aerobic physical activity combined with adequate muscle-strengthening activity and agreeing with the statement that electronic social welfare and healthcare services help in maintaining a healthy lifestyle.

Previously, eHealth use has been reported more common among women [30,32], which is not supported by this population survey study conducted in the Finnish context. In China, the price of a WT device has been found to be irrelevant to the users’ adoption of fitness WT [12], which is in line with our finding that experiencing financial difficulties is not connected to WT use. BMI was not a relevant factor for WT use, even though WT use was associated with exercising more regularly. A similar finding has been detected earlier [42], and generally, it is believed that weight loss and weight maintenance are inadequate without a complementary diet [43]. WT use has also been found meaningful for weight loss in another study, whereas a healthy diet seemed to require counselling on health habits [26]. Even though there were considerable differences between the groups who agreed and did not agree with electronic services providing support for maintaining a healthy lifestyle, the variable was not statistically significant for WT use. The findings seem consistent with prior research as diet is not central to most WT devices, whereas exercising is [3]. Wynn et al. [44] have demonstrated that a high education level is associated with eHealth use. However, education level was not a statistically significant factor for WT use, which gives a slight hope that WT use is more equal than other forms of eHealth services. Analysing the association between WT and quality of life or mental well-being would require a more in-depth study design.

### Older adults (65–99)

Older adults tend to track health related information because they find the information useful, they intend to share it with a healthcare professional, or they want to comply with their healthcare provider’s instructions [23]. WT for activity monitoring has been recognized as a potential tool for supporting a healthy lifestyle in the ageing population [16]. In our study, marital status, getting adequate aerobic physical activity weekly, and independent e-service use were found to be positively associated with WT use among older adults. Older adults’ health conditions have previously been demonstrated to influence technology adoption [46], which possibly leads to a situation where healthier people start using WT more often compared to people with disabilities or functional health deficits. In our study older adults of all other marital status groups used WT markedly more often compared to singles. Considering means to motivate older adults living alone and those with chronic conditions and disabilities to use WT might be meaningful since regular exercising has a positive effect on health.

According to the Technology Acceptance Model (TAM), intention to use technology is dependent on its’ ease of use and usefulness. These factors then can further be influenced by moderating individual factors and factors rising from other theories [34]. For example, the self-assessed level of digital skills has been shown to decrease with age [30] and as a rule, age has been shown to play a major role in interaction with technology [34]. In this study, WT use decreased with age, and while age was associated with WT use in the general adult population, it was not among older adults. WT users seemed to have better digital skills, since older adults who used e-services independently used WT 15 times more often compared to those who did not use e-services independently or at all. Therefore, the lack of necessary digital skills may act as a significant barrier to WT use. The adoption of new technologies has in general been observed to face resistance in the ageing population, which has been explained by the new technologies conflicting with the prevailing socio-technical regime of older adults [16]. On the other hand, providing older adults information of the option of WT use and its’ possible benefits have been stressed by other studies [22,33].

A scoping review identified the lack of self-efficacy, knowledge, support, and understanding of possible benefits as key barriers to e-health engagement [45]. It has been suggested that to invite older adults to use technologies that support their welfare, the technology should be easy to use, but also available and adaptable to the users’ daily lives while being compatible with their needs [46]. The usability of a WT device has among older adults been shown to act as a determinant of satisfaction with the device [31]. On the other hand, older adults do not form a homogenous group and their needs should be assessed individually while at the same time considering possible obstacles to adopting new technology [47]. Providing users customizable tracker designs has been suggested by researchers to be meaningful for the continued use of WT in all ages [14].

### Reliability of the results and future research themes

The response rate of the survey was 46.5 percent, which means that over half of the target group did not respond to the survey. However, the reliability of the results was enhanced by the robust population survey data set. Analysis weights based on register data were used in the analysis to correct the effects of non-response. The study utilized established independent variables that provide credibility for the results. The use of WT can be short-lived for many users [10], which might affect the respondents’ evaluation of whether they had or had not used WT in the last 12 months. Therefore, the results reflect the respondents’ decision to report use or non-use at the time of answering.

In the future, utilizing a longitudinal approach, studying the effects of specific WT devices, and conducting qualitative research would be beneficial. Qualitative research that provides understanding of the context of use and the needs of the users could help produce applicable results for decision-making and development purposes as well as promote designing user-oriented WT for people of all ages. The negative association between WT use and sleep also requires a more detailed study approach. In the future, it would be important to shift the research focus to the quality of long-term measurement trends and the scope and process (e.g., ease of interpretation of measurement trends) of WT application in healthcare in order to support the introduction of WT to patient care safely. In connection to this, the research of WT use calls for a systematic approach for assessing the possible positive and negative effects of use. Building upon this study, research should next focus on user characteristics and characteristics of non-users of WT devices used for more specific purposes (e.g., WT for weight maintenance) to enable these more in-depth study designs in the future.

### Conclusions

This study sheds light on factors that are associated with WT use in the Finnish adult population (20–99) and among older adults (65–99). Compared to previous studies, this study analyses the associations in a large, representative sample covering people aged 20 to 99 years old. The results may be used as a reference, when planning future studies on the subject and by companies designing WT devices. WT users in the Finnish adult population and among older adults exercised more compared to non-users. WT use was more common among younger respondents when the whole adult population was examined, but in the data for older adults age did not play a role. WT use was also more common among persons who were married, cohabiting, separated, divorced, or widowed compared to those who were single. Among the adult population, WT use was associated with not sleeping enough and agreeing with that using social welfare or healthcare e-services help in taking an active role in looking after ones’ own health and well-being. There was also a slight association with a self-assessed good state of health. Among older adults, WT use was strongly associated with independent e-service use. WT use was not associated with sex, education level, mental health, quality of life, BMI, or agreeing with that using social welfare or healthcare e-services help in maintaining a healthy lifestyle.

Among older adults WT use was non-existent when the respondent did not use e-services independently, which raises the question whether the needs of older adults and the elderly should be better considered in the design of WT devices. The results concerning the more active lifestyle of WT users show potential from the perspective of public health. However, this study was based on a large, cross-sectional population survey that analysed associations between WT use and sociodemographic, well-being, health, benefit, and lifestyle variables. Since the data was not produced for studying the effects of WT use, the possible positive impact on users’ activity level needs to be researched more thoroughly before WT can be considered a tool for tackling the challenges of the prevailing sedentary lifestyle.

## Materials and methods

### The study samples

This cross-sectional study was a national population survey (The National FinSote Survey) carried out by the Finnish Institute for Health and Welfare. The data collection took place during the COVID-19 pandemic from September 2020 to February 2021. The survey focused on the health, well-being and service use of adults aged 20 and older. All respondents first received a postal survey from Finnish Institute for Health and Welfare, which included the questionnaire and a pre-paid envelope for returning the questionnaire and a printed link to the online version of the survey. The survey could be answered in Finnish, Swedish, English, or Russian. An ethics committee approval (THL/637/6.02.01/2017) was received from the Ethics Committee of the Finnish Institute for Health and Welfare. Potential participants were informed of their rights in a cover letter and in a privacy notice, and they provided an informed consent to take part in the study by deciding to answer the questionnaire: ‘By responding to the survey, I agree that my personal data will be processed in accordance with the privacy statement and that my responses may be combined with health and well-being register data.’

The main survey questionnaire (sample size n = 61,600, respondents n = 28,199, response rate after removal of over-coverage 46.4%) covered general health, well-being, and service use. A sub sample got an additional digi-module (sample size n = 13,200, respondents n = 6,034, response rate after removal of over-coverage 46.5%) that concentrated on social welfare and healthcare digitalization and e-services. The digi-module was designed in 2014 and updated for this survey round. The new questions concerning WT and app use were designed by a research group including two researchers of this study (MK, TV). No WT companies were involved in the study process.

The sampling method was a stratified random sampling design with 22 strata, one for every well-being services county in Finland. The sampling was executed in the Finnish Population Information System. In every well-being services county, the sample size was 2,800 (2,000 in the age group of 20–74-year-olds and 800 in the age group of over 74-year-olds). In the digi-module, the sample size was 600 in every well-being services county (400 in the age group of 20-74-year-olds and 200 in the age group of over 74-year-olds) [48].

In this study, the used data set (questions of the main survey and the digi-module) included respondents from the digi-module sample (n = 6,034). In the analysis, non-response was corrected using analysis weights based on register data and produced with the Inverse Probability Weighting (IPW) method, which has been shown to improve the accuracy of results in similar studies [49]. The final model (sex, age, marital status, education, residential area, and language) of IPW was selected using Bayesian information criterion [48].

### Variables

The outcome variable in the study was ‘have you during the last 12 months used wearable technology such as a fitness tracker or a smart ring to support activity, well-being or a healthy lifestyle’ (no/ no but I would be interested/ yes). As background information, the prevalence of smart technology use that supports independent living, such as a smart security bracelet or device that automatically calls for help, was also reported. Missing values in the outcome variable were coded as non-users to enhance the relevance of the results. The demographics included sex (register-based), age (register-based), education level, marital status, and financial difficulties. The lower age-limit (65) of the older adult group was defined based on the prevalence of WT use in five-year-age-groups in the data and based on 65 being a general retirement age in Finland. The study sample did not have an upper age limit, but in practice the oldest respondents were 99 years old.

The education level (low, middle, high) variable was based on the question “How many years altogether have you attended school or studied full time? Including primary and comprehensive school ____ years.” The respondents were first divided into 10-year age groups by sex. After this, terciles of years of education were calculated in each group and the educational level was defined using those terciles as cut-off points. Financial difficulties were measured by three questions (yes/no): In the past 12 months, have you ever (a) feared that you will run out of food before you can get money to buy more? (b) been unable to buy medicines because you did not have any money and (c) not visited a doctor because you did not have any money. Answering yes to one or more of the questions indicated financial difficulties.

Well-being scores included two scales and one question, which express the respondents’ subjective self-assessments. Quality of life was measured on an eight-item scale: the World Health Organization Quality of Life (WHOQOL-8) scale [50]. The respondents’ average scores were divided into two groups: below and above the sample average. The second scale was the 7-item Short Warwick-Edinburgh Mental Well-being Scale [51]. The scorings were divided as follows: 7–20 probable or possible depression and 21–35 average or high mental well-being. The respondents were asked to describe their state of health as good, fairly good, average, fairly poor or poor.

Lifestyle and risk factors were analysed using five variables. Aerobic physical activity accounted for 150 minutes of moderate-intensity or 75 minutes of vigorous-intensity aerobic physical activity weekly. Aerobic and muscle-strengthening physical activity included the previously mentioned exercise and muscle-strengthening or muscle-perpetuating activities on two or more days a week. The latter activity amount is currently recommended in Finland. Sleep, a crucial factor for daily activity and time-use in general [52], was examined with one question: Do you feel that you get enough sleep (yes-almost always; yes-often; rarely or hardly ever; not sure)? BMI was calculated based on respondents’ weight and height. Two categories of BMI were used: equal to or above 25 and equal to or above 30.

Benefits were measured with two questions: How do you feel about the following claims concerning the benefits of electronic social welfare and healthcare services? They help me to maintain a healthy lifestyle; They help me to take an active role in looking after my own health and well-being (completely agree, somewhat agree, neither agree nor disagree, somewhat disagree, strongly disagree). E-service use was also researched among older adults since it has been shown to be rarer in the older age groups [30]: Do you use the Internet for the following: e-services (e.g., My Kanta Pages, MyTax, the Social Insurance Institution of Finland [Kela]). The response options were ‘I use it independently’, ‘I use it with another person’s help or someone else uses it on my behalf’ and ‘I don’t use it’.

### Statistical analysis

The data was processed with IBM SPSS Statistics Version 27 using complex sample methods designed for analysing data with statistical weights. In the first table (Table 1) the prevalence of wearable technology use was examined in the adult population grouped by socio-demographic, well-being, benefit, and lifestyle factors. Logistic regression models were constructed to analyse the associations between WT use and factors for both among the adult population (Table 2 and Table 3) and among older adults (Table 4 and Table 5). The analysis was adjusted for older adults so that the age groups were smaller, BMI of 25 or over was excluded, and e-service use was added to the model. The model selection was done by reviewing the results of the Wald test: non-statistically significant variables (p>0.05) were excluded. Multicollinearity was tested in RStudio Version 4.2.2 using variance inflation factor (VIF) measure. The small VIF values indicated low correlation between the models’ variables that is acceptable in logistic regression analysis. All analyses were conducted using statistical weights.
